# Generation of Rational Drug-like Molecular Structures Through a Multiple-Objective Reinforcement Learning Framework

**DOI:** 10.3390/molecules30010018

**Published:** 2024-12-24

**Authors:** Xiangying Zhang, Haotian Gao, Yifei Qi, Yan Li, Renxiao Wang

**Affiliations:** Department of Medicinal Chemistry, School of Pharmacy, Fudan University, 826 Zhangheng Road, Shanghai 201203, China

**Keywords:** molecular generative model, de novo drug design, multi-objective optimization, GCPN

## Abstract

As an appealing approach for discovering novel leads, the key advantage of de novo drug design lies in its ability to explore a much broader dimension of chemical space, without being confined to the knowledge of existing compounds. So far, many generative models have been described in the literature, which have completely redefined the concept of de novo drug design. However, many of them lack practical value for real-world drug discovery. In this work, we have developed a graph-based generative model within a reinforcement learning framework, namely, METEOR (Molecular Exploration Through multiplE-Objective Reinforcement). The backend agent of METEOR is based on the well-established GCPN model. To ensure the overall quality of the generated molecular graphs, we implemented a set of rules to identify and exclude undesired substructures. Importantly, METEOR is designed to conduct multi-objective optimization, i.e., simultaneously optimizing binding affinity, drug-likeness, and synthetic accessibility of the generated molecules under the guidance of a special reward function. We demonstrate in a specific test case that without prior knowledge of true binders to the chosen target protein, METEOR generated molecules with superior properties compared to those in the ZINC 250k data set. In conclusion, we have demonstrated the potential of METEOR as a practical tool for generating rational drug-like molecules in the early phase of drug discovery.

## 1. Introduction

Virtual screening of compound libraries has been a widely adopted approach in structure-based drug discovery for finding novel lead compounds. However, the potential exploration of molecules with desired properties is severely curtailed by the limited size of available compound libraries (~10^9^) [1]. This constraint pales in comparison with the vast chemical space of “drug-like” compounds, which is estimated to range from 10^23^ to 10^60^ [2]. To bridge this gap, de novo drug design offers another approach to delving into the chemical space beyond existing compounds. Conventional de novo design methods typically rely on a pre-defined fragment library to construct molecular structures in a stepwise manner. Such a building-up process is relatively time-consuming, and yet the structural diversity among the generated molecular structures is in principle limited by the fragment library employed therein. Moreover, conventional de novo design methods often produce molecular structures that are challenging to synthesize due to extensive enumeration [3,4]. All these obstacles have hindered the wide application of de novo design to practical drug discovery efforts.

In recent years, generative models, a type of unsupervised training model, have emerged as invaluable tools in various scientific domains [5]. Such models have been able to generate new samples by comprehending the essential probability distribution underlying the given training samples. Generative models quickly found their applications in the realm of chemistry, where they were typically trained on large compound libraries to capture the intrinsic probability distribution embedded in the molecular structures. By drawing samples from the learned distribution, novel molecular structures were generated, which effectively expanded the accessible chemical space. It has been demonstrated that even a tiny fraction, for example, 0.1%, of a compound library, when used to train a generative model, could cover a significant portion of the chemical space spanned by the entire library [6]. Thus, generative models hold great promise in expanding the arsenal for drug discovery. Particularly for de novo drug design, generative models can not only create new molecules but also to craft molecules of specific interest.

Reinforcement learning presents an approach for achieving targeted molecule generation [7]. Within the framework of reinforcement learning, an agent engages with an environment through a sequence of actions. The agent iteratively refines its policy to maximize cumulative rewards across the action sequence, guided by the environment’s feedback. In the context of de novo drug design employing reinforcement learning, an environment is tailored to provide rewards to the agent based on the properties of the generated molecules. Previous studies have demonstrated the utility of reinforcement learning in biasing generative models toward the creation of molecules with desired optimized properties [8,9,10,11,12,13,14,15,16,17,18].

However, many of the current generative models exhibit certain limitations when being evaluated in real-world drug discovery scenarios. For example, some models aim at overly contrived objectives, such as maximization of log *P* without any limit [8,9,12,17,19]. Some other models focus exclusively on the binding affinity against a specific target [10,11,14,15,16]. However, a successful drug discovery process is multi-objective in nature, where one has to consider and evaluate multiple properties of the candidates simultaneously [20,21,22,23]. Therefore, we believe that a generative model with practical value for de novo drug design has to be trained in a multi-objective manner.

Accordingly, we have developed such a molecular generative model, namely, METEOR (Molecular Exploration Through multiplE-Objective Reinforcement). METEOR is integrated with a reinforcement learning framework, which allows the rapid design of molecules with desirable drug-likeness and synthetic accessibility, as well as binding affinity to a user-defined target protein. In METEOR, we employ the Graph Convolutional Policy Network (GCPN) originally proposed by You et al. [8] as the fundamental architecture to construct the backend generative model. We evaluated several graph traversal algorithms [24] in terms of their efficiency in molecular structure generation. We also introduced chemical rules to detect improper substructures, thereby substantially elevating the quality of the molecular structures generated. Importantly, we introduced a special reward function to promote multi-objective optimization, combining considerations of binding affinity to the target protein, drug-likeness, and synthetic accessibility. Here, binding affinity to the target protein was evaluated by PLANET, a GNN-based deep learning model developed by our group [25]. Finally, we showcased the potential application of METEOR to real-world drug discovery with a retrospective example.

## 2. Results and Discussion

### 2.1. Comparison of Generative Models Based on Different Algorithms

The several generative models developed in our study were trained on the ZINC 250k data set in order to generate valid molecular graphs. To evaluate the performance of these models in this aspect, metrics encompassing validity, uniqueness, and novelty were considered. These metrics were assessed based on a sample of 50,000 molecules generated by each model.

The validity of the generated molecules remained at 100% across all models (Table 1), which should be attributed to the step-by-step valency check enabled during graph generation. In contrast to SMILES-based models, which might encounter validity issues due to syntax problems, graph-based generative models benefit from a more natural representation of molecular structures, ensuring high validity. However, a validity check by RDKit does not guarantee drug-like molecular structures (Figure S1 in the Supporting Information). Thus, we have implemented additional chemical rules in the molecular generation environment in our model to filter out undesired substructures, including cumulative alkenes and peroxyl bonds, double or triple bonds in three or four-membered rings, bridged rings formed with aromatic rings, and large rings. Our analysis indicated that these additional substructure detections led to the elimination of approximately 40% of the impractical structures generated by GCPN_origin_. Moreover, the breadth-first model (BFM) was observed to have a problem with ring closure (Figure S2 in the Supporting Information). This problem arose due to the divergent nature of breadth-first graph generation, where the generative model tended to generate molecular graphs with incomplete rings, which may be closed later after several inconsecutive actions. In contrast, GCPN and the depth-first model (DFM) in principle can generate molecular graphs with rings in a more practical and complete manner.

Among our evaluation metrics, uniqueness reflects the fraction of non-duplicate molecules, while novelty reflects the fraction of generated molecules not presented in the training set. Our results show that BFM exhibits the lowest performance in terms of uniqueness and novelty (Table 1). By analyzing BFM-generated molecules, we have observed that the graph generation process is prone to terminate prematurely and produce simple and duplicate structures. In order to evaluate the scaffold uniqueness and novelty presented by the molecules in the ZINC 250k data set, we extracted the Bemis–Murcko scaffolds for all of them. Our results revealed that GCPN_ours_ and DFM achieve similar metrics, while the performance of BFM is limited by its preference for molecules with simple structures.

**Figure 1:**
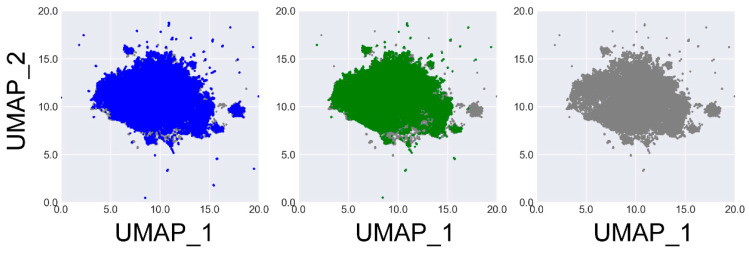
UMAP projection of the ZINC 250k molecules (grey) and those generated by DFM (blue) and GCPN_ours_ (green), respectively. This plot illustrates the similarity between the chemical spaces covered by different generative models.

To gain a deeper understanding of the chemical space covered by the molecules generated by these several generative models, we performed UMAP projection on the molecules generated by GCPN_ours_ and DFM, as well as 50,000 molecules randomly selected from the ZINC 250k data set. Here, UMAP analysis was performed with the umap-learn Python package [26]. Molecules were represented by their the extended-connectivity fingerprints (ECPF4) fingerprints hashed to 1024 bits. The resulting binary vectors were then reduced to 250 dimensions using principal component analysis before being projected onto two dimensions. The results are illustrated in Figure 1. One can see that both the outcomes given by GCPN_ours_ and DFM effectively span the chemical space represented by the training set (i.e., ZINC 250k), indicating their comparable ability to generate diverse molecule structures.

### 2.2. Test Case: De Novo Design with METEOR

As discussed above, GCPN_ours_ and DFM demonstrated a remarkable advantage over BFM, this test attempted to evaluate the performance of METEOR_GCPN_ and METEOR_DFM_ within the realm of reinforcement learning. We then wanted to examine their performance in a real de novo drug design scenario. The objective here was to design ligand molecules targeting glucocerebrosidase (GBA), simultaneously optimizing essential properties including drug-likeness, synthetic accessibility, and binding affinity to the target.

**Figure 2:**
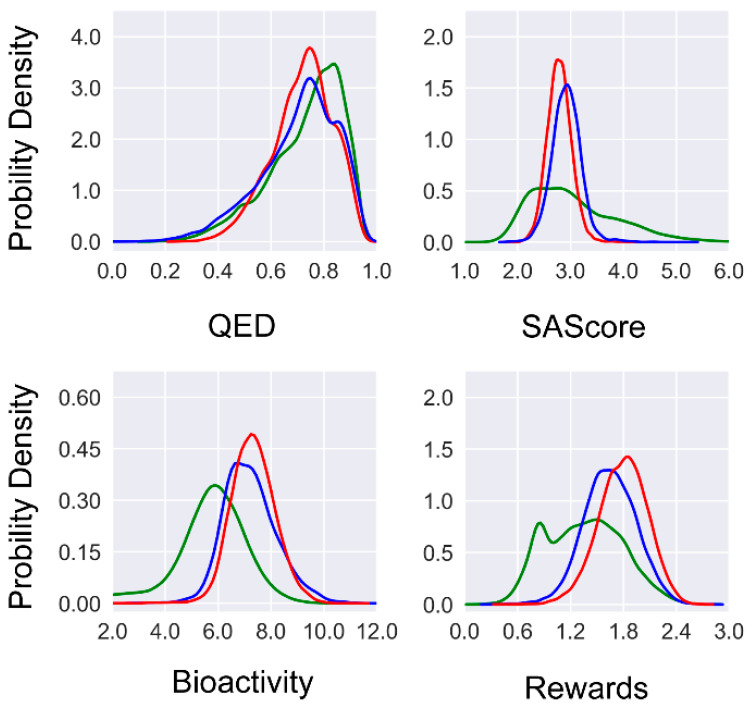
Distribution of three desired features and the unweighted sum of rewards of the molecules generated at the last round of reinforcement learning (red lines: METEOR_GCPN_; blue lines: METEOR_DFM_; green lines: ZINC 250k).

To investigate the effect of multi-objective optimization, we examined the three desired features (i.e., drug-likeness, synthetic accessibility, and binding affinity) of the molecules generated at the initial round and the final round of reinforcement learning (Figure 2). Firstly, a notable improvement in the predicted binding affinity can be observed if comparing the molecules generated by METEOR_DFM_, METEOR_GCPN_, and those from ZINC 250k. Regarding the QED value, a significant fraction of the molecules generated by METEOR_DFM_ and METEOR_GCPN_ (77.6% and 83.8%, respectively) exceeded the QED threshold of 0.6. Nevertheless, no notable improvement in the QED value was observed after reinforcement learning. This is because the ZINC 250k data set as a whole already exhibits a high level of QED value, leaving very limited room for further improvement. Regarding the SAScore value, the majority of ZINC 250k molecules fall within the range of (1.5, 5.0). After reinforcement learning, SAScore of the generated molecules concentrated at the range of (2.5, 3.5) with a more focused distribution. Furthermore, both models generated fewer molecules that were predicted to be challenging for synthesis as compared to the ZINC 250k molecules. Considering all three features together, the distribution of the unweighted sum of three feature rewards shifted to the right as compared to the distribution of the ZINC 250k molecules. To conclude, both METEOR_DFM_ and METEOR_GCPN_ were able to generate novel molecules with improved predicted binding affinity under the constraints of drug-likeness and synthetic accessibility.

### 2.3. METEOR_GCPN_: Has a Larger Action Space as Well as a Higher Learning Efficiency

**Figure 3:**
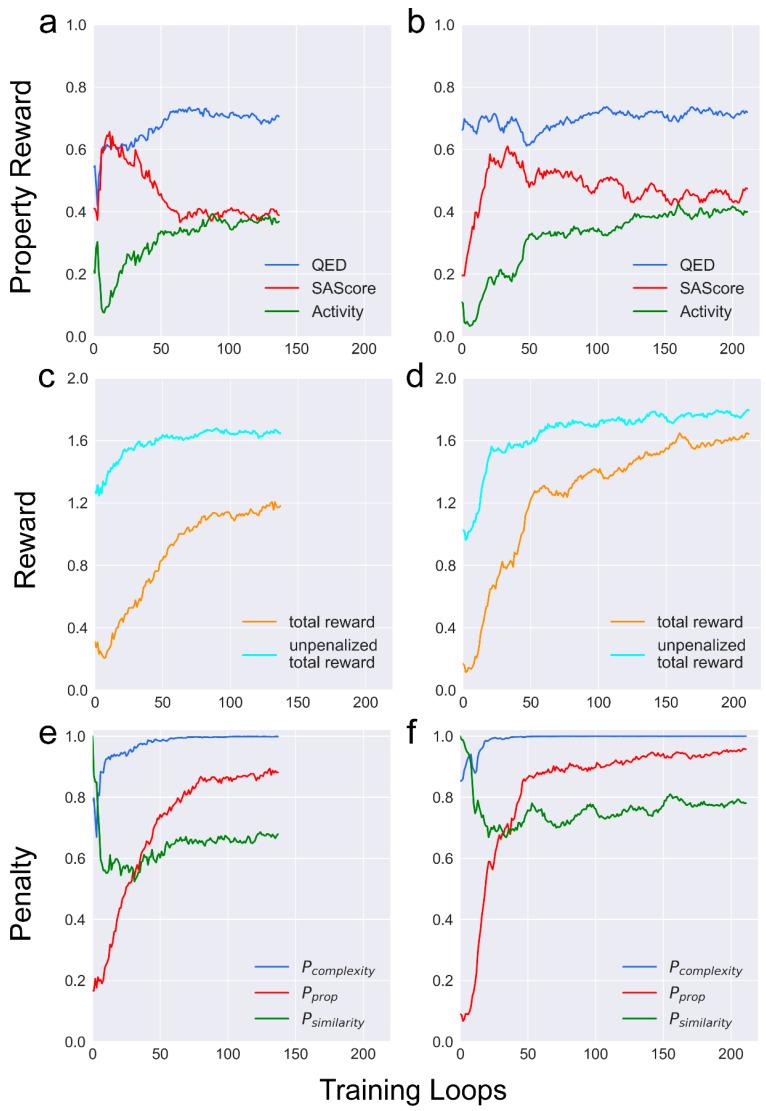
Several key features of METEOR_DFM_ (**left**) and METEOR_GCPN_ (**right**) observed during the reinforcement learning process. (**a**,**b**): Three property rewards; (**c**,**d**): Total and unpenalized rewards; (**e**,**f**): Three penalty factors. Here, each line plots the mean value of a certain feature computed over all molecules generated at each round of roll-out.

Incorporating the depth-first graph traversal algorithms in DFM eliminates the need to decide the starting atom for adding a new bond. This modification reduces the action space of METEOR_DFM_ and theoretically streamlines reinforcement learning. However, the learning curve demonstrated that METEOR_GCPN_ can be trained at a higher level of stability and efficiency than METEOR_DFM_ in reinforcement learning (Figure 3a,b). After 50 rounds of reinforcement learning, METEOR_GCPN_ received a mean total reward of around 1.25, whereas the mean total reward of METEOR_DFM_ at the same point was approximately 0.85. Despite the smaller action space of METEOR_DFM_, the full trajectory for METEOR_DFM_ for generating a molecular graph is roughly twice as long as that of METEOR_GCPN_. This inequality accounts for the different efficiency of METEOR_DFM_ and METEOR_GCPN_. For example, over a three-day period of reinforcement learning, METEOR_GCPN_ generated around 2.7 million molecules across 212 rounds, whereas METEOR_DFM_ generated around 1.8 million molecules over 138 rounds. Given the same amount of training time, the additional training iterations achieved by METEOR_GCPN_ make it possible to uncover molecules with improved properties. Moreover, GCPN’s inherent capability of determining when to terminate the graph expansion allows METEOR_GCPN_ to assess the attributes of the existing molecular structure. This capability empowers METEOR_GCPN_ to judiciously halt the expansion of a graph when the current structure exhibits particularly favorable attributes. This explains why METEOR_GCPN_ generated molecules with superior synthetic accessibility in comparison to METEOR_DFM._

In addition, a notable disparity was observed between the total reward and the unpenalized reward acquired by both METEOR_GCPN_ and METEOR_DFM_ (Figure 3c,d). This gap primarily arose from the property penalty at the early training phase (Figure 3e,f). In METEOR, property penalty (Equation (5)) was the driving force for multi-objective optimization on binding affinity to the protein, drug-likeness, and synthetic accessibility. Computing rewards by a weighted sum across three property rewards reinforced the optimization to be conducted toward all three properties.

**Figure 4:**
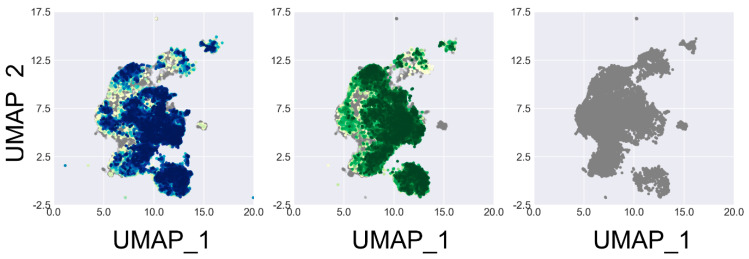
UMAP projections of 50,000 ZINC 250k molecules (grey) and 50,000 molecules generated at certain rounds by METEOR_DFM_ (blue) and METEOR_GCPN_ (green), respectively. Different rounds are indicated in colors with different shades.

The complexity penalty was computed primarily by counting heavy atoms. This penalty was introduced to balance the bias along structure generation, where larger molecules tend to receive higher predicted binding scores by PLANET. Moreover, larger molecules often contain challenging moieties for chemical synthesis, such as chiral centers. The similarity penalty was introduced to encourage METEOR to explore the chemical space preventing it from becoming confined to local maxima. During the initial training rounds, a relatively modest similarity penalty was observed among the molecules generated by both METEOR_DFM_ and METEOR_GCPN_ due to the presence of limited high-scoring molecules recorded in the memory stack. At the 40th round or so, the influence of similarity penalties became more obvious (Figure 3e,f). Here, both METEOR_DFM_ and METEOR_GCPN_ were able to explore the full chemical space covered by the ZINC 250k data set throughout the training process without being trapped in certain restricted regions (Figure 4).

In conclusion, both METEOR_DFM_ and METEOR_GCPN_ are able to generate molecules with optimized properties. The major distinction between METEOR_DFM_ and METEOR_GCPN_ lies in their efficiency.

### 2.4. The Practical Value of METEOR in De Novo Drug Design

**Figure 5:**
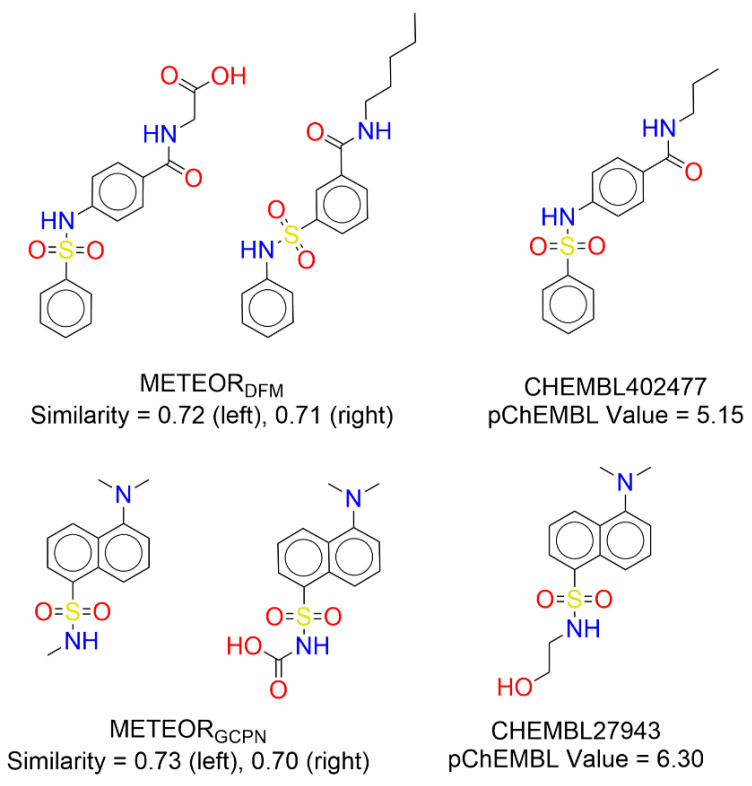
Examples of the molecules generated by METEOR_DFM_ and METEOR_GCPN_ as well as the corresponding true binders of GBA.

In this study, we evaluated the practical value of METEOR in de novo drug design by using GBA as a test case. The quality of the molecules generated by METEOR was reflected by analyzing their similarity to true binders of GBA collected from ChEMBL. If using an ECFP4 Tanimoto coefficient of 0.6 as the threshold, 15 molecules generated by METEOR_DFM_ shared similar structures to true binders to GBA. As for METEOR_GCPN_, this number was 17. A few such examples are given in Figure 5. One can see that the generated molecules shared an almost identical scaffold as a certain GBA binder. This observation demonstrated that METEOR is able to generate drug-like molecules with potential value.

It should be mentioned though that as a whole, a substantial proportion of the true GBA binders considered in our study have a QED value below 0.5 or SAScores over 3.5 (Figure S3 in the Supporting Information). However, the majority of the molecules generated by METEOR had optimized QED values and SAScores that do not stay at this range (Figure 2). Thus, in this particular test cast, this gap resulted in rather limited matched pairs between the outcomes of METEOR and true GBA binders.

Moreover, we employed the GLIDE module in the Schrödinger software, a widely used conventional molecule docking method, to evaluate the binding affinity of the molecules generated by METEOR with the target protein. Prior to the molecular docking job, the molecules generated during the reinforcement learning process in METEOR were filtered based on the following criteria: (1) an QED value above 0.6, (2) an SAScore lower than 3.0, and (3) a predicted binding affinity value greater than 7.0 (in -log units). The molecules meeting all requirements were then docked into the binding pocket on GBA. To make a comparison, all ZINC 250k molecules were also docked into the binding pocket on GBA through the same protocol.

**Figure 6:**
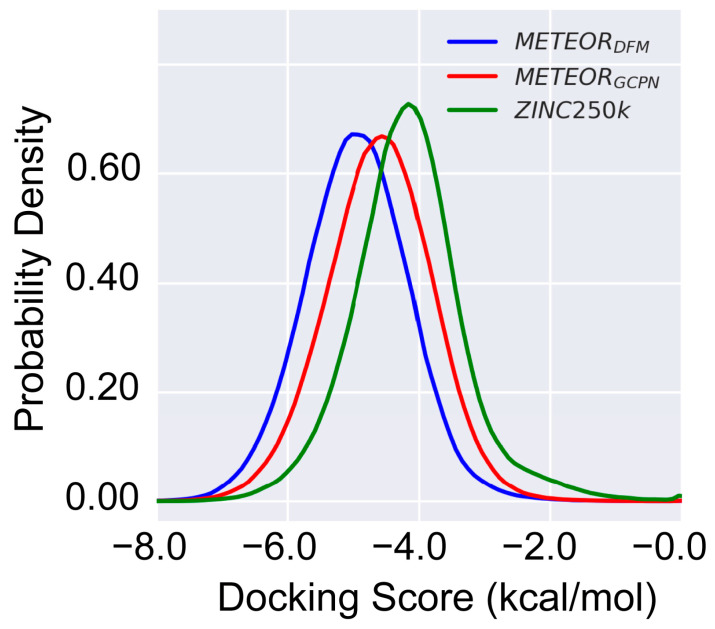
Distribution of the GLIDE docking scores computed for several sets of molecules: ZINC 250k molecules (*n* = 247168, green), METEOR_DFM_ molecules (*n* = 279910, blue), and METEOR_GCPN_ molecules (*n* = 739130, red).

As shown in Figure 6, the molecules generated by either METEOR_GCPN_ or METEOR_DFM_ on average had better GLIDE binding scores than those ZINC 250k molecules, even though even though the optimization of binding affinity in METEOR was guided by a different scoring function PLANET. The 1% percentile of docking scores was −6.20, −6.83, and −6.57 for molecules from ZINC 250k, METEOR_DFM_, and METEOR_GCPN_, respectively. Note that besides binding affinity to the target protein, the molecules generated by METEOR were also optimized in terms of drug-likeness and synthetic accessibility. Therefore, it is reasonable to expect that more promising active hits can be discovered through application of METEOR rather than a conventional virtual screening of the ZINK 250k data set.

## 3. Methods

### 3.1. The Backend Molecule Generative Models

**Figure 7:**
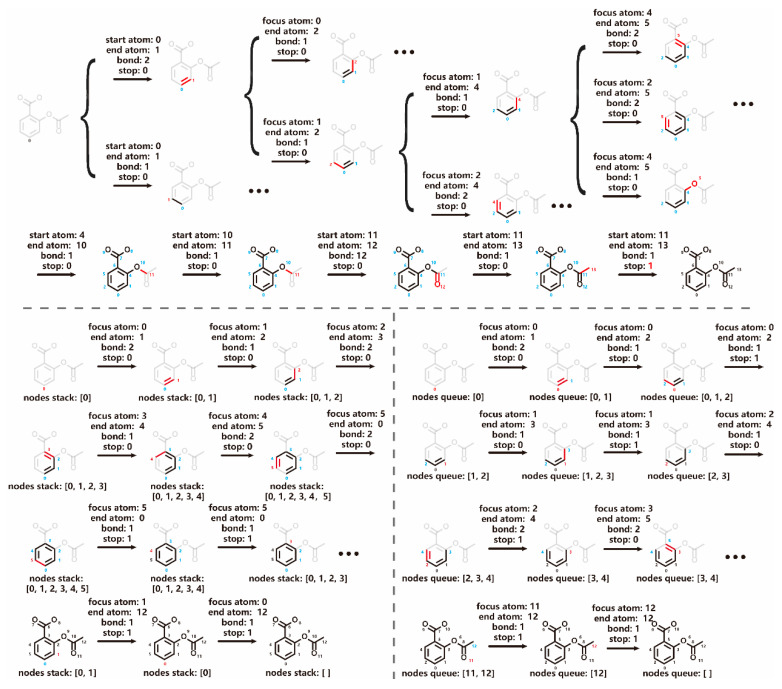
Illustration of graph generation process for the aspirin molecule by GCPN (**top**), DFM (**bottom left**, in a depth-first manner), and BFM (**bottom right**, in a breadth-first manner). At each step, the end atoms of new bonds are marked in red, possible focus atoms are marked in blue, and “finished atoms” are depicted in black.

The GCPN model extends an existing molecular graph by adding new chemical bonds one after another (Figure 7). During this process, four decisions need to be made at each step: (1) determining the starting atom (“focus atom”) to which the new bond is added, (2) selecting the end atom of the new bond, (3) specifying the type of the new bond, and (4) deciding whether to terminate graph expansion [8]. The first decision significantly expands the action space of GCPN, leading to numerous possibilities within each existing subgraph. To address this complexity, we introduced two models: DFM and BFM, each employing a distinct graph traversal algorithm (Figure 7). In both models, the “focus atom”, defined by the respective graph traversal algorithm, serves as the starting point for adding a new bond. In alignment with DFM and BFM, the final task in GCPN, i.e., determining whether to terminate graph generation, is replaced by marking the current focus atom as “finished”. Graph generation terminates when all nodes have been marked.

Individual atom nodes were encoded using vectors with a dimension of 20, consisting of one-hot encoded element type, atom degree, and membership in rings of varying sizes from 3 to 7. These initial vectors were then embedded into a latent space ($h_{0}$) with a size of 64 dimensions. To extract features from the input graphs, we utilized the graph convolution network (GCN) architecture [27,28,29]. The entire molecular graph was partitioned into three distinct subgraphs based on different bond orders. One module with three separate graph convolution layers with a hidden size of 64, each with learnable parameters $W_{i}^{l}$, were employed on the three subgraphs, as denoted in Equation (1).

(1) $$h^{l}=\sum_{i=1}^{b}\left(ReLU\left({\tilde{D}}_{i}^{-\frac{1}{2}}{\tilde{A}}_{\dot{i}}{\tilde{D}}_{i}^{-\frac{1}{2}}h^{\left(l-1\right)}W_{i}^{l}\right)\right),b\in \left\{1,2,3\right\}$$

$A_{\dot{i}}$ is the *i*th slice bond-conditioned adjacent matrix, ${\tilde{A}}_{\dot{i}}$ = $A_{\dot{i}}+I$; ${\tilde{D}}_{i}$ is the *i*th slice bond-conditioned degree matrix with self-loop.

In our implementation, we utilized three such modules to extract the underlying features from the molecular graphs. The extracted latent features were subsequently utilized to make informed decisions within the model. The configuration of task layers in GCPN remained consistent with the original literature [8]. In the case of DFM and BFM, when selecting ending atom for the chemical bond to be added, nodes marked as “finished” were overlooked. The probability of each action $P\left(a_{t}\right)$ was calculated as shown in Equation (2):

(2) $$P\left(a_{t}\right)=\left(1-I\left(a_{t}^{finish}\right)\right)\prod_{j}P\left(a_{t}^{j}\right)+I\left(a_{t}^{finish}\right)P\left(a_{t}^{finish}\right)$$

Each action step $a_{t}$ is composed of three sub-tasks $a_{t}^{j}$, i.e., selecting the end atom of the new bond, specifying type of the new bond, and whether to mark the focus atom as “finished”. $I\left(a_{t}^{finish}\right)$ equals 0 if the focus atom is not decided to be marked, else $I\left(a_{t}^{finish}\right)$ equals 1.

To enhance stability and performance in reinforcement learning, a commonly employed strategy involves pre-training a generative model using an established compound database [30]. In our study, we employed the widely-used ZINC 250k data set, comprising structurally diverse “drug-like” molecules that have been synthesized in reality. Structures of these molecules were examined to eliminate those containing rings with eight or more members. The remaining molecules were considered as the ground truth and served as expert training data. For GCPN pre-training, a randomly sampled connected subgraph G’ from a molecule graph G was viewed as the state $s_{t}$. Any action $a_{t}$ added an atom or a bond in G but not in G’ could be viewed as an expert action during the trajectory of generating a ground-truth molecule. The training objective was to maximize the possibility $P\left(a_{t}\right)$ of GCPN to take expert action $a_{t}$ at state $s_{t}$. This training approach was similar to that previously reported [8]. For both DFM and BFM, the molecular structures from the filtered ZINC 250k data set were transformed into expert trajectories by randomly selecting a starting node and traversing the graphs in a depth-first or breadth-first manner, respectively. The resulting expert actions $a_{t}$ consisting of the trajectories were collected for pre-training DFM and BFM. The objective in expert training for all models can be expressed as shown in Equation (3):

(3) $$L^{expert}\left(\theta \right)=-\frac{1}{T}\sum_{t}^{T}logP\left(a_{t}\right)$$

The Adam optimizer with a learning rate of 0.0001 was applied. After 1,000,000 training steps, GCPN, DFM, and BFM with converged loss were obtained as pre-trained generative models.

### 3.2. The Molecule Generation Environment

Within the context of molecular graph generation under a reinforcement learning framework, the molecule generation environment plays two essential roles, i.e., state transition dynamics and reward assignment.

#### 3.2.1. State Transition Dynamics

The molecule generation environment plays a pivotal role in executing the actions taken by the agent, ensuring adherence to specified rules. One fundamental rule incorporated into the environment is the valency check, preventing actions that exceed an atom’s maximal valency [8]. It is noteworthy that substructures adhering to the basic valency rule may not be “drug-like”. Therefore, the environment in our model detects and then filters out the following “non-drug-like” substructures: (a) cumulative alkenes and peroxyl bonds; (b) double or triple bonds in a three- or four-membered ring; (c) bridged ring formed with aromatic rings; and (d) large rings with eight or more members, as detected in the smallest set of smallest rings in a molecular graph. Substructure detection is performed after each agent action through SMARTS matching (see Figure S4 in the Supporting Information). Only actions that pass both the valency check and substructure examination will be adopted by the environment to update the current molecule subgraph. Note that implementation of the above chemical rules reflects the knowledge of “drug-likeness” accumulated in the literature [31,32,33]. There are of course different perceptions of “drug-likeness”, but the several rules listed above are relatively straightforward to be encoded in a computer program. In particular, macrocyclic structures are not allowed in our model, although some marketed drugs do consist of such structures [34]. From a practical view, macrocyclic structures are normally introduced at the stage of lead optimization to impose conformational constraints. Considering that our model will be employed primarily as an “idea generator” at the stage of lead discovery, ignoring macrocyclic structures is an acceptable trade-off for the sake of technical convenience.

#### 3.2.2. Reward Assignment

The behavior of agents is steered by the rewards from the molecule generation environment, which can be categorized into two components: step reward and final reward. A zero-step reward is assigned to each step, except for two specific actions: (a) When a new ring is formed, a small step reward of 0.02 is assigned to encourage ring formation. (b) When an improper action is canceled by the molecular generation environment, a step reward of −0.2 is given to discourage such actions. The step reward serves to guide the agent’s behavior and reduce the occurrence of improper actions. The final reward comprises several domain-specific rewards assigned based on different properties, including drug-likeness, synthetic accessibility, and predicted bio-activity. The final reward is calculated as the weighted sum of these rewards, further adjusted by a penalty factor. Reward functions related to specific properties utilize a linear scaling function that maps values between a lower bound and an upper bound, as described in Equation (4):

(4) $$R_{prop}=\left\{\begin{array}{cc} 1.0 & \;S_{prop}\geq S_{prop}^{high}\;;\\ \frac{S_{prop}-S_{prop}^{low}}{S_{prop}^{high}-S_{prop}^{low}}, & \;S_{prop}^{low}<S_{prop}<S_{prop}^{high};\\ 0.0 & \;S_{prop}\leq S_{prop}^{low}\;. \end{array}\right.$$

This type of function is chosen based on the assumption that it is not necessary to optimize certain properties beyond desired ranges. For example, it is not necessary to further optimize the synthetic accessibility of a molecule with an SAScore lower than 2.0 because it is already good enough at this level.

Generated molecules are evaluated by the following three properties:

(a) Drug-likeness of a molecule is assessed by the QED index originally proposed by Hopkins et al. [33]. This index has a range (0.0, 1.0).

(b) Synthetic accessibility of a molecule is evaluated by SAScore, which has a range [1.0, 10.0]. SAScore is a rule-based tool for estimating synthetic accessibility, and its output is determined by the summation of fragment scores and a complexity penalty [35].

(c) Binding affinity to the target protein is predicted by PLANET, a graph neural network model developed in our group [25]. PLANET operates on two-dimensional molecular graphs as inputs and thus skips the exhaust molecular docking process. Its ultra-fast speed is suitable for processing generated molecules in a large number.

A penalty factor is also implemented to influence the agent model’s behavior by scaling the sum of property rewards. This factor is determined based on three aspects:

(a) Complexity penalty ($P_{complexity}$). The complexity penalty is assigned based on the number of heavy atoms in the designed molecule, defined as a linear scaling function akin to Equation (4). The lower and upper bounds for the number of heavy atoms are set to 10 and 40, respectively. Additionally, for molecules with more than two chiral centers, a penalty factor of 0.5 will multiply $P_{complexity}$.

(b) Property penalty ($P_{prop}$). Since the reward is the sum of three property rewards, it is possible for an agent to receive a high reward from a molecule that possesses two excellent properties but one extremely poor property. The property penalty is applied as follows (Equation (5)):

(5) $$P_{prop}=\prod_{prop}\min\left(1.0,R_{prop}/0.2\right)$$

(c) Similarity penalty ($P_{similarity}$). Agents trained in reinforcement learning tend to generate highly-scored molecules. However, once a local maximum is reached, agents often struggle to explore other areas, leading to a phenomenon known as “policy collapse”. Inspired by the work of Blaschke et al. [14], we devised a similarity penalty to encourage agents not only to focus on specific favorable regions in the chemical space yielding high scores but also to explore various areas within the space. Our algorithm for calculating the similarity penalty differs from that of Blaschke though. For example, all halogen atoms are ignored here to prevent our model from generating molecular structures with differences merely in the number and position of halogen atoms. This is important since at the stage of lead discovery, sufficient diversity in the structural scaffold is much desired, where terminal halogen atoms are not part of a structural scaffold. In fact, halogen atoms are often added to optimize bioactivity at a later stage of drug discovery. Subsequently, a mapping between the current molecule and those generated before the preceding twenty rounds of roll-out is performed. If a successful mapping is found, a zero-penalty factor is assigned. Molecules passing this mapping step proceed to subsequent similarity calculation. A stack is used to retain favorable molecules, that is, those generated over the preceding 10 rounds of roll-out with a final property reward surpassing 70% of the possible maximum. Tanimoto similarity coefficients between the ECFP4 of the transformed molecule and all stored high-quality molecules are calculated. $P_{similarity}$ is determined based on the maximal Tanimoto similarity coefficient, as outlined in Equation (6):

(6) $$P_{similarity}\left\{\begin{array}{cc} 0.0 & \;\mathrm{Success}\;\mathrm{Mapping}\;\mathrm{OR}\;\mathrm{Tanimoto}\geq 0.7\;;\\ 1-(\mathrm{Tanimoto}-0.4)/0.3, & \;0.4<\mathrm{Tanimoto}<0.7\;;\\ 1.0 & \;\mathrm{Tanimoto}\leq 0.4\;. \end{array}\right.$$

The final reward ($R_{final}$) is the weighted sum of all property rewards scaled by overall penalty (Equation (7)):

(7) $$R_{final}=\sum^{i}\alpha_{i}R_{i}\times \prod^{j}P_{j}$$

Here, *i* and *j* denote for different types of molecular properties and penalty factors, respectively.

#### 3.2.3. Reinforcement Learning

Policy gradient-based methods are widely adopted in reinforcement learning. In our model, Proximal Policy Optimization (PPO) is adopted [36]. The learning objective can be written as Equation (8):

$$L_{PPO}^{\theta^{k}}\left(\theta \right)=-\sum_{\left(s_{t},a_{t}\right)}min\left(\frac{P_{\theta }\left(a_{t}|s_{t}\right)}{P_{\theta }^{k}\left(a_{t}|s_{t}\right)}A^{\theta^{k}}\left(a_{t},s_{t}\right),clip\left(\frac{P_{\theta }\left(a_{t}|s_{t}\right)}{P_{\theta }^{k}\left(a_{t}|s_{t}\right)},1-\varepsilon ,1+\varepsilon \right)A^{\theta^{k}}\left(a_{t},s_{t}\right)\right)$$

(8) $$A^{\theta^{k}}\left(a_{t},s_{t}\right)=\sum_{t=t^{\prime }}^{T_{n}}\gamma^{T_{n-}t^{\prime }}R_{final}+R_{step}-b$$

In the objective function, γ represents the discount factor, and its value is experimentally set to 0.98. The clip value, denoted as ε, is set to 0.1. The superscript *k* denotes for the generative model obtained after the last round of training. The estimated advantage function $A^{\theta^{k}}\left(a_{t},s_{t}\right)$ incorporates a learnable value function *b*. The value function takes the same molecular graph embedding obtained from the GCN layers and maps it to a scalar representing the estimated expected reward. The probability of an action taken by the generative model with parameter θ under state $s_{t}$, denoted as $P_{\theta }(a_{t}|s_{t})$, is calculated using Equation (2).

### 3.3. Performance Evaluation

#### 3.3.1. Evaluation in Terms of Generating Valid Molecular Structures

We assessed the performance of several pre-trained generative models, including the GCPN_origin_ (only enabling valency check during graph generation), the GCPN_ours_, DFM, and BFM (all three utilizing full substructure check, including valency check and improper substructure detection, see Figure S4 in the Supporting Information). Each pre-trained model was assigned the task of generating 50,000 molecules for evaluating validity, uniqueness, and novelty as follows:

$$Validity=\frac{Number\;of\;valid\;graphs}{Number\;of\;generated\;graphs}$$

$$Uniqueness=\frac{Number\;of\;unique\;and\;valid\;graphs}{Number\;of\;valid\;graphs}$$

$$Novelty=\frac{Number\;of\;unique\;and\;valid\;graphs\;not\;in\;the\;training\;set}{Number\;of\;unique\;and\;valid\;graphs}$$

Valid graphs were typically measured with respect to valency and bonds using RDKit’s molecular structure parser.

#### 3.3.2. Evaluation in Terms of Generating Useful Hits on a Specific Target Protein

To evaluate the effectiveness of METEOR in generating useful hits in a de novo drug design scenario, we chose GBA as the target protein, which is included in the popular LIT-PCBA benchmark for testing virtual screening methods [37]. The crystal structure of GBA used in our test is obtained from the Protein Data Bank (PDB entry 2V3D) [38]. Two pre-trained generative models, namely, GCPN_ours_ and DFM, served as backends of METEOR (denoted as METEOR_GCPN_ and METEOR_DFM_, respectively, hereafter). Major adjustable parameters in these models are in Equation (4), where the lower bound of QED, SAScore, and binding affinity was set to 0.2, 3.5, and 5.5, respectively, and the upper bound was set to 0.8, 2.0, and 8.5, respectively.

Our test was performed on a server equipped with two NVIDIA GeForce 2080Ti GPU cards (11 GB memory), two Intel(R) Xeon(R) Silver 4210 CPUs @ 2.20 GHz, and 128 GB of RAM. After three days of reinforcement learning with 10 parallel processes, all generated molecules were assessed in two aspects: Firstly, a total of 452 true binders of GBA were curated from ChEMBL, which were identified by a “Target ChEMBL ID” of CHEMBL2179 and a “pChEMBL value” greater than 5.0 (for example, *K*_d_ or *K*i value < 10 μM). Pairs of molecules with an ECFP4 Tanimoto coefficient over 0.6 were defined as similar. The total number of molecules generated by METEOR that were similar to true binders to GBA was counted and analyzed. Secondly, the molecules generated by METEOR, filtered based on QED value, SAScore, and binding affinity, were docked into the binding pocket of GBA by using GLIDE in the standard precision (SP) mode in the Schrödinger software. To make a comparison, the molecules in the ZINC 250k data set were docked into the binding pocket of GBA following the same protocol.

## 4. Conclusions

In this work, we have developed a deep learning mode, called METEOR, for potential application in de novo drug design. Compared to many other generative models already described in the literature, METEOR has several distinct technical features.

Firstly, the backend agent of METEOR is based on the well-established GCPN model. We have evaluated several graph traversal algorithms within a reinforcement learning framework. Our findings indicate that depth-first graph generation (DFM) outperforms breadth-first graph generation (BFM). Its outcomes closely align with those of the original GCPN model in terms of validity, uniqueness, and novelty. This observation supports the potential value of both METEOR_GCPN_ and METEOR_DFM_ in de novo drug design. As demonstrated in the test case of GBA, without prior knowledge of true binders, both models are able to generate molecules with superior properties compared to those in the ZINC 250k data set.

Secondly, in order to ensure the overall validity of the generated molecular structures, we have implemented a set of chemical rules in METEOR to eliminate undesired substructures. In fact, if these rules are not enabled, a significant portion (~40%) of the generated molecule structures would be undesirable. This demonstrates the importance of integrating chemical knowledge into molecular structure generation, which has become a new trend in this field (for example, see a new generative model published recently [39]).

Last, and very importantly, unlike many other generative models that focus on a single objective, METEOR is designed to generate molecules with optimized traits regarding binding affinity, drug-likeness, and synthetic accessibility. These several properties are all indispensable for a successful candidate in the early phase of drug discovery. This makes METEOR better suited for practical applications to drug discovery.

## Figures and Tables

**Table 1:** Metrics of 50,000 molecules generated by several generative models.

Models	Validity			Uniqueness		Novelty	
	RDKit	Pattern	Completeness	Molecule	Scaffold	Molecule	Scaffold
GCPN (origin)	1.000	0.592	0.993	1.000 ^a^	0.666 ^a^	1.000 ^a^	0.928 ^a^
GCPN (ours)	1.000	1.000	0.960	1.000	0.737	1.000	0.953
DFM	1.000	1.000	0.987	0.912	0.626	0.999	0.914
BFM	1.000	1.000	0.677	0.776	0.454	1.000	0.925

^a^: Molecules containing improper substructures are viewed as invalid.

## Data Availability

Source code of METEOR, as well as user manual and demo examples, are available at https://github.com/ComputArtCMCG/METEOR (accessed on 12 December 2024).

## Supplementary Materials

The following supporting information can be downloaded at: https://www.mdpi.com/article/10.3390/molecules30010018/s1, Figure S1: Examples of valid but unwanted molecular structures; Figure S2: Examples of molecules with “ring closure” issue; Figure S3: Distribution of QED, SAScore and PLANET binding scores of the known GBA binders collected from ChEMBL; Figure S4: Valency check and improper substructure detection implemented in different generative models.
