# Supplementary material for: Generation of Rational Drug-like Molecular Structures Through a Multiple-Objective Reinforcement Learning Framework
Source: Molecules. 2024 Dec 24;30(1):18. doi: 10.3390/molecules30010018 (PMC11721775; doi:10.3390/molecules30010018)
Supplement: Supplementary file 1 [file molecules-30-00018-s001.zip › molecules-3342857-supplementary.pdf]

Supporting Information

Generation of Rational Drug-Like Molecular Structures Through a Multiple-Objective Reinforcement Learning Framework

Xiangying Zhang, Haotian Gao, Yifei Qi, Yan Li,* and Renxiao Wang*

Department of Medicinal Chemistry, School of Pharmacy, Fudan University, 826 Zhangheng Road, Shanghai 201203, People's Republic of China

* To whom all correspondence should be addressed: *li_yan@fudan.edu.cn* (Y. Li); *wangrx@fudan.edu.cn* (R. Wang)

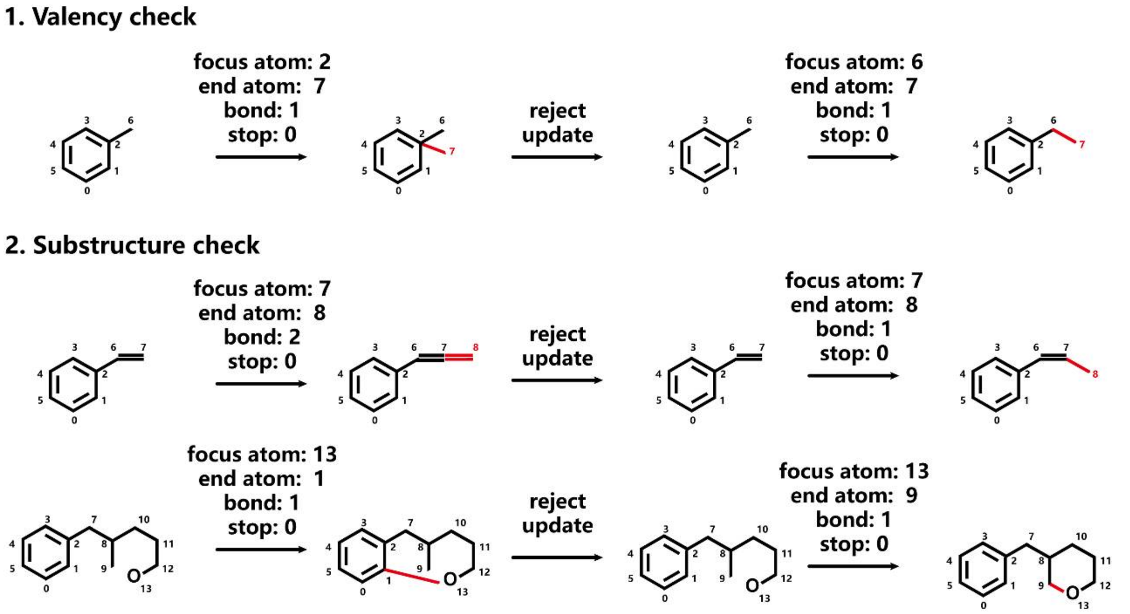

Figure S1. Valency check are enabled in GCPN$_{origin}$, GCPN$_{ours}$, DFM, and BFM, but substructure check is disabled in GCPN$_{origin}$. SMARTS for detecting not allowed substructures include “*=[#6]=*”, "[#8]-[#8]", "[#16]-[#16]", "[#9,#17,#35,#53]=,#* “a1(*2)a@2aaaa1”, “a1@2aa@1aaa@2”, “a1(*2)aaaa@2a1”,”a1(**2)aaaa@2a1”, “*1(*2)=**=*@2*=*1”, “a1(**2)aaa@2aa1”, “a1(***2)aaa@2aa1”, “a1(*2)a@2aaa1”, “a1@2aa@1aa2”, “a1(*2)aa@2aa1”, “a1(**2)aa@2aa1” and “a1(***2)aa@2aa1”.

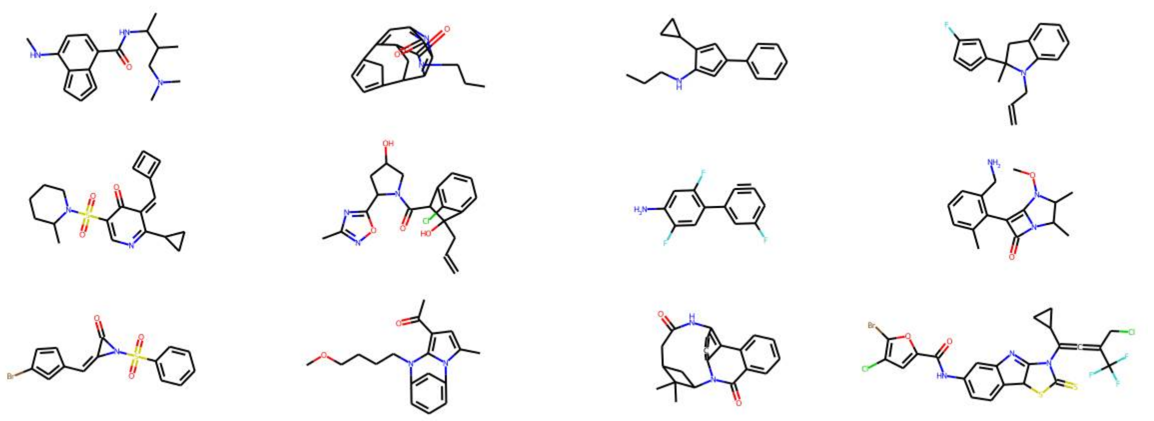

Figure S2. Examples of molecular structures that do not violate the valency rules by RDKit exmination, but are obviously not desired in drug design.

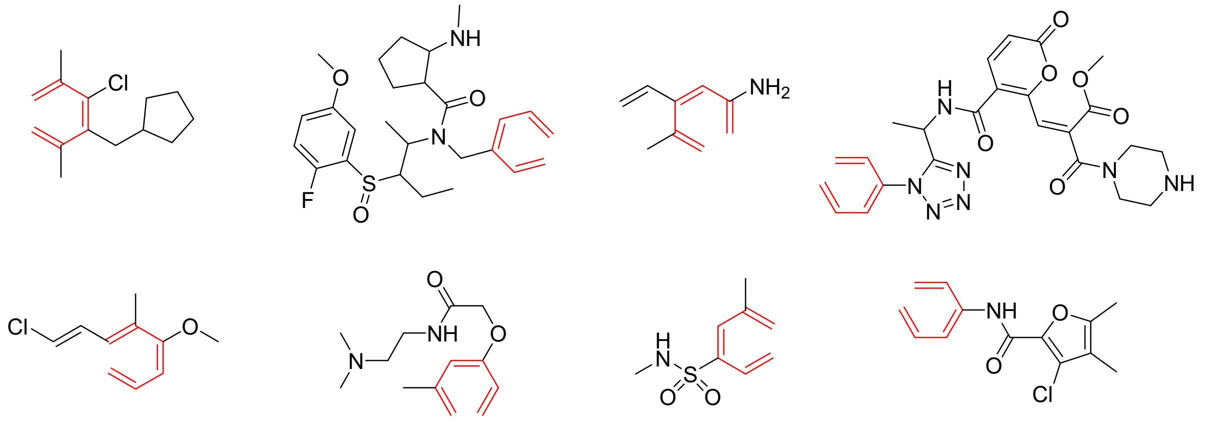

Figure S3. Examples of molecules with "ring closure" issue. "Unclosed" substructure is highlighted.

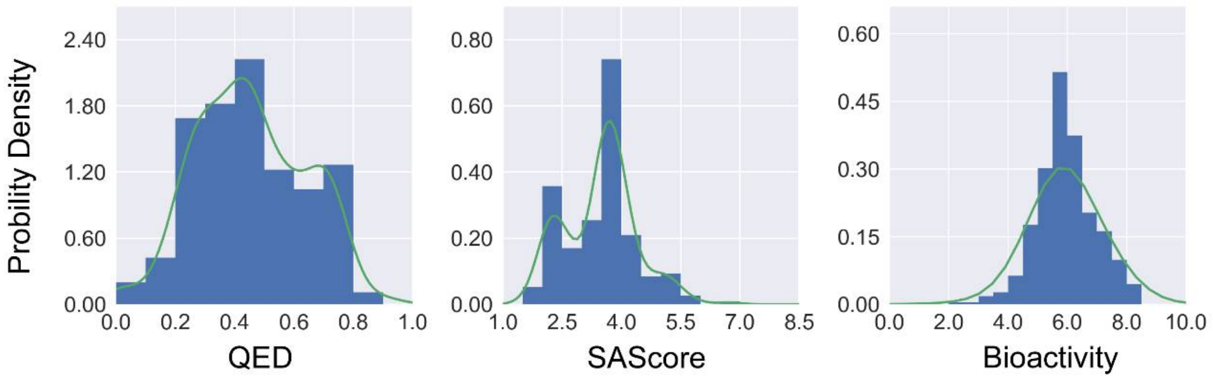

Figure S4. Distribution of QED, SAScore and PLANET binding scores of the known GBA binders collected from ChEMBL (N = 452).
